# Health Diplomacy in Africa: Prospects, Obstacles, and the Way Ahead

**DOI:** 10.1002/puh2.70245

**Published:** 2026-05-01

**Authors:** Majani Edward

**Affiliations:** ^1^ Research and Training Unit, Department of Research and Innovation MacWish College of Health and Allied Sciences Mwanza Tanzania; ^2^ Centre for Reforms, Innovation, Health Policies and Implementation Research Dodoma Tanzania

**Keywords:** Africa, global health governance, health diplomacy, health systems strengthening, regional collaboration

## Abstract

**Introduction:**

Africa faces a complex and evolving health landscape marked by endemic diseases, emerging infectious threats, fragile health systems, climate change, and geopolitical pressures. In this context, health diplomacy has become an important mechanism for fostering collaboration, mobilizing resources, and advancing collective health security.

**Objective:**

This review examines the prospects and obstacles of health diplomacy in Africa and proposes strategic directions to enhance its effectiveness in achieving equitable and sustainable health outcomes.

**Methods:**

A narrative review was conducted using a purposive search of literature published between 2016 and 2026 across PubMed, Scopus, and Google Scholar, complemented by policy documents and institutional reports. A total of 152 records were identified, with 43 sources included after screening. Data were analyzed through thematic synthesis, guided by decolonial theory and liberal institutionalism to interpret governance dynamics.

**Results:**

Key prospects include strengthened regional collaboration through African Union mechanisms, improved bargaining power in global health governance, increased engagement of non‐state actors, and the integration of digital health innovations. However, significant obstacles persist, including political diversity, sovereignty concerns, resource inequalities, weak institutional capacity, coordination gaps, and external influence. Historical power asymmetries continue to shape negotiation processes and health governance structures.

**Conclusion:**

Health diplomacy offers a critical pathway to address shared health challenges in Africa. Strengthening its impact requires investment in diplomatic capacity, policy alignment, digital innovation, and equitable partnerships, supported by a unified and equity‐driven continental approach.

## Abstract 2

This article examines the role of health diplomacy in addressing Africa's complex health challenges. It identifies key opportunities, including regional collaboration, digital innovation, and strengthened global engagement, alongside persistent barriers such as resource inequalities, political diversity, and institutional weaknesses. The findings highlight the need for coordinated policies, capacity building, and equitable partnerships to enhance the effectiveness of health diplomacy in achieving sustainable health outcomes.

## 1 Introduction

Africa's health landscape is characterized by a complex interplay of endemic diseases, emerging infectious threats, AMR, climate change, persistent conflicts, and persistent healthcare system weaknesses [1]. In this context, health diplomacy emerges as a crucial instrument for fostering collaboration and achieving collective health goals across the continent. Building upon the understanding of global health diplomacy [2], health diplomacy in Africa can be defined as the strategic engagement and negotiation among African states, regional bodies, non‐state actors, and international partners to address shared health challenges, harmonize health policies, and strengthen health systems for the benefit of all African people [3]. This approach recognizes the unique political, economic, and social dynamics of the continent and seeks to leverage diplomatic tools to overcome obstacles and capitalize on existing and emerging opportunities in the health sector [1]. The urgency of this matter is particularly acute in the African context. The continent faces a persistent triple burden: endemic diseases (malaria, HIV/AIDS, tuberculosis), the constant threat of high‐impact emerging and re‐emerging infectious diseases (Ebola, Mpox), and foundational weaknesses in health infrastructure, funding, and skilled human resources [4, 5]. This situation is significantly compounded by increasing regional interconnectedness (fueled by expanding trade, migration, and shared porous borders), which ensures that health challenges rapidly transcend national boundaries. This reality underscores a fundamental principle: Unilateral actions are insufficient; collaborative and diplomatic solutions are indispensable for achieving collective health security and well‐being [1, 6]. This article aims to provide a comprehensive analysis of the opportunities that health diplomacy presents for Africa, the significant challenges that must be navigated to realize its full potential, and the key future directions that can guide its evolution for a healthier and more secure Africa.

## 2 Methodology

This study employed a narrative review design to synthesize existing literature on health diplomacy in Africa. A narrative approach was chosen due to its suitability for examining complex, multidisciplinary issues that intersect global health, international relations, and development studies.

A purposive literature search was conducted covering the period from 2016 to 2026 to capture recent developments in health diplomacy across the African context. Searches were performed in major academic databases, including PubMed, Scopus, and Google Scholar, alongside relevant policy documents and institutional reports from key organizations, such as the World Health Organization (WHO), African Union (AU), and Africa Centres for Disease Control and Prevention (Africa CDC).

The search strategy utilized combinations of key terms, such as “health diplomacy Africa,” “global health governance Africa,” “regional health cooperation Africa,” “health security Africa,” and “African health governance.” The initial search yielded 152 records comprising peer‐reviewed articles, policy reports, book chapters, and institutional publications. Following title and abstract screening for relevance, 70 sources were selected for full‐text review, of which 43 met the inclusion criteria and were included in the final synthesis.

Inclusion criteria encompassed sources addressing health diplomacy or global health governance within African settings, with a focus on regional or international cooperation, policy negotiation, power dynamics, or health system strengthening. Studies focused exclusively on clinical or biomedical topics without relevance to governance or diplomacy were excluded.

Data were analyzed using thematic synthesis, enabling the identification and organization of key patterns into three analytical domains: (1) prospects for health diplomacy in Africa, (2) structural and political obstacles, and (3) strategic directions for strengthening health diplomacy. The analysis was further informed by decolonial theory and liberal institutionalism, providing a framework to interpret the influence of historical power asymmetries, institutional collaboration, and global governance structures on health diplomacy in Africa.

## 3 Conceptual Framework

Various international relations theories can help explain how Africa, particularly in limited resource setting, can progress toward real health diplomacy [7]. Among these, decolonial theory provides an important perspective for analyzing health diplomacy efforts in Africa [8]. To apply this lens, one must first understand colonialism, which has been defined as “*one group of people having the power to dominate another group or groups of people, thereby enabling the misappropriation and extraction of resources in a large‐scale and systematic manner”* [9]. A decolonial perspective offers a way to understand and challenge how these inherited power dynamics are created and upheld, thereby influencing health diplomacy in Africa. These dynamics manifest in three forms of power essential to understanding participatory governance: visible, hidden, and invisible. Visible power relates to what can be observed. Hidden power refers to powerful people and institutions that maintain their influence by controlling who gets to the decision‐making table and what gets on the agenda. Finally, the most pervasive form is invisible power [10].

Several scholars argue that hidden power often operates within international relations, enabling dominant actors to shape the agenda priorities [8, 10]. This underlying hidden power frequently rooted in colonial histories sustains dependency [3]. The argument emphasizes that these colonial histories are not merely leftovers from the past but are still actively upheld today through modern mechanisms, such as corporate and financial colonialism [8].

In practice, mitigating dependency requires strategies that emphasize robust cooperation and the strengthening of the regional health system. Health diplomacy, as the art and practice of conducting negotiations, can be an effective tool to decolonize hidden power in the interrelationships among actors [11]. Diplomats working within the negotiation system respect established processes and agreed methods to reach a compromise and consensus. This ultimately upholds a legitimate order, which provides the context for diplomacy [6].

In the 21st century, health has undergone a significant transformation. It is a key aspect of geopolitics, indicating that it is no longer just “low politics” but a core part of global foreign policy. It is operationalized through distinct frames: security, development, trade, global public goods, human rights, and ethical reasoning [7, 12]. As Labonté and Gagnon observe, the “high politics” of health security and trade often overshadow the “low politics” of ethics and human rights, which generally lack enforcement mechanisms [12]. Health priorities are influenced by social language and norms, not just facts. To advance equity, diplomats should act as norm entrepreneurs—for example, framing health as a fundamental human right rather than a charitable act [13]. For diplomats operating in Africa, understanding this distinction is a strategic advantage, allowing them to employ “smart power” strategies that link health initiatives to broader security and development goals. However, relying solely on the health security frame can be risky, as it may reflect policymakers’ priorities—such as infectious threats—rather than epidemiological or community‐driven needs, such as strengthening hospitals. This narrow approach risks undermining local ownership and sustainability, and perpetuating power imbalances [12].

Furthermore, to ensure resources lead to long‐term resilience rather than short‐term projects, we must draw on liberal institutionalism. This perspective emphasizes that, in an interdependent world, a sustainable path involves robust cooperation and the strengthening of national health systems [7].

During the COVID‐19 pandemic, countries engaged in two different types of health diplomacy: One aimed at promoting solidarity and equity, and the other at pursuing geopolitical interests and advantage [4].

**FIGURE 1:**
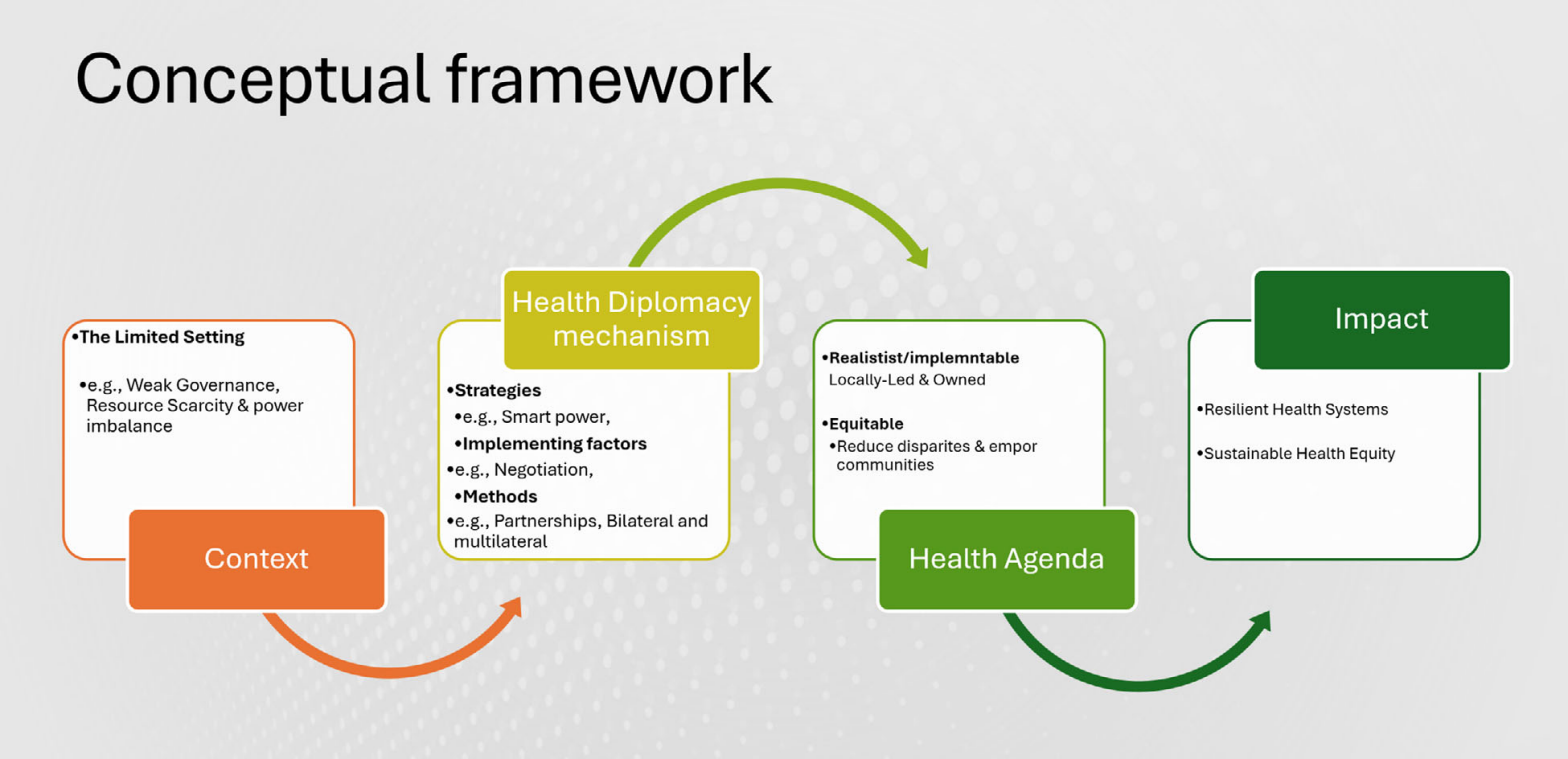
Conceptual framework (health diplomacy in Africa). *Source:* Prepared by the author for this publication.

Building on this insight, this article proposes a conceptual framework that integrates structural conditions, diplomatic strategies, and health governance outcomes (Figure 1). The framework draws on insights from decolonial theory and liberal institutionalism to explain how historical power relations and institutional cooperation shape health diplomacy processes.

First, the structural context reflects the broader political and economic environment in which health diplomacy occurs. Many African countries operate in settings characterized by limited financial resources, fragile health systems, and historical power asymmetries rooted in colonial legacies [11]. From a decolonial perspective, these dynamics influence who sets global health agendas, whose knowledge is prioritized, and how resources are distributed within international health governance [7].

Second, within this structural context, diplomatic strategies and mechanisms are deployed by states and institutions to advance health priorities. These strategies include multilateral negotiations, regional policy coordination, coalition‐building among states, partnerships with non‐state actors, and regulatory innovation. Health diplomacy therefore extends beyond traditional foreign policy to involve ministries of health, regional organizations, civil society actors, and global health institutions.

Third, the framework highlights implementation outcomes, which represent the tangible results of diplomatic engagement. These include improved regional cooperation, strengthened health systems, increased access to medicines and technologies, and greater African influence in global health governance.

Figure 1 illustrates how these three components interact. Structural conditions shape the opportunities and constraints for diplomatic engagement, whereas diplomatic strategies mediate these conditions to generate policy outcomes.

## 4 Prospects

Africa possesses a wealth of opportunities to harness the power of health diplomacy for significant advancements in public health. One prominent opportunity lies in strengthened regional collaboration and institutional integration [3]. The role of AU and its specialized public health agency, Africa CDC, provides a compelling case study. During the COVID‐19 pandemic, Africa CDC coordinated pooled procurement of diagnostics and personal protective equipment through the Africa Medical Supplies Platform [14, 15]. This collective diplomatic engagement enabled member states to negotiate better prices and secure access to scarce commodities in a highly competitive global market. The initiative demonstrated how unified continental diplomacy can mitigate market inequities and strengthen collective bargaining power. The existence of strong regional economic communities (RECs), like the AU, Economic Community of West African States (ECOWAS), SADC, and the East African Community (EAC), provides established frameworks for diplomatic engagement on health issues [14]. These platforms can facilitate the harmonization of health policies, joint surveillance initiatives, and coordinated responses to cross‐border health threats. Leveraging these existing structures can streamline diplomatic efforts and build upon established relationships and shared objectives [2]. For example, the ECOWAS has long coordinated cross‐border surveillance and epidemic preparedness through the West African Health Organization (WAHO). Following the 2014–2016 Ebola outbreak, ECOWAS member states strengthened collaborative disease surveillance mechanisms and emergency response coordination [11, 16]. This regional diplomatic engagement reduced fragmentation and fostered shared accountability in managing health security threats that transcend national borders.

Furthermore, Africa has a significant opportunity to strengthen its collective bargaining power in the global health arena. By speaking with a unified voice on issues, such as access to essential medicines, technology transfer, and research funding, African nations can exert greater influence in international negotiations and secure more favorable outcomes. Health diplomacy can facilitate the development of common positions and coordinated advocacy strategies to amplify Africa's voice on the global stage [3]. A notable case is the continent's unified advocacy for vaccine equity during the COVID‐19 pandemic. African leaders, through the African Vaccine Acquisition Trust (AVAT), negotiated directly with vaccine manufacturers to secure doses for member states [17]. Simultaneously, South Africa and India championed the TRIPS waiver proposal at the World Trade Organization, advocating temporary intellectual property flexibility to expand vaccine production in low‐ and middle‐income countries [18]. Although negotiations were complex and outcomes partial, this episode illustrated Africa's increasing engagement in shaping global trade‐health intersections and challenging structural inequities in access to essential medicines.

The continent also has a burgeoning opportunity to leverage its growing engagement with non‐state actors. African civil society organizations, NGOs, and philanthropic foundations possess deep community knowledge and play crucial roles in health service delivery and advocacy [13]. A notable example is the partnership between African governments and the Bill & Melinda Gates Foundation in malaria control programs, demonstrating how diplomatic alignment can mobilize substantial financial and technical resources [19]. These initiatives are not merely technical collaborations but reflect diplomatic engagement that aligns national health priorities with global philanthropic investments. Through coordinated negotiations and strategic engagement, several African countries have scaled up malaria interventions, strengthened surveillance systems, and improved access to preventive tools. This case highlights how diplomacy extends beyond intergovernmental negotiation to include strategic alliance‐building with global philanthropy and civil society. Engaging these actors in health diplomacy processes can bring valuable perspectives, enhance community buy‐in, and ensure that health policies are responsive to the needs of the population. Similarly, responsible engagement with the private sector can unlock innovation and resources for health development [1].

Harnessing digital health technologies presents another significant opportunity for health diplomacy in Africa. The rapid advancements in mobile technology and digital platforms offer unprecedented opportunities for cross‐border information sharing, telemedicine, and remote monitoring [13]. Diplomatic collaborations can facilitate the development of interoperable digital health systems, the establishment of data governance frameworks, and the sharing of best practices in utilizing technology to improve health outcomes across the continent [20]. Rwanda provides a notable example through its partnership with technology companies to deploy drone‐based medical supply delivery systems in collaboration with Zipline [21]. Beyond technological innovation, this initiative illustrates how health diplomacy can facilitate cross‐sector collaboration between governments, private technology firms, and regulatory institutions. Through strategic diplomatic engagement, the Rwandan government established an enabling regulatory environment that allowed unmanned aerial delivery systems to operate within national airspace. This process required negotiation between health authorities, aviation regulators, and international technology partners, demonstrating how diplomatic coordination and regulatory innovation can accelerate access to essential medical supplies in remote settings. The initiative therefore highlights how health diplomacy can support technological innovation while ensuring national ownership and policy oversight.

Moreover, Africa could learn from and adapt successful health diplomacy models from other regions while tailoring them to its unique context. Studying how other regional blocks have addressed shared health challenges through diplomatic engagement can provide valuable insights and lessons learned. South–South cooperation with other developing regions facing similar health issues can also offer opportunities for knowledge exchange and collaborative initiatives [22].

A notable milestone is Africa's experience with the establishment of the African Medicines Agency (AMA), which represents a landmark diplomatic achievement [23]. Negotiated under the AU framework, the AMA treaty aims to harmonize medicine regulation across member states, improve quality assurance, and strengthen pharmaceutical manufacturing capacity within the continent. This initiative reflects how sustained diplomatic negotiations can translate political commitments into institutional mechanisms that advance long‐term health sovereignty and regional integration.

Finally, the increasing global focus on health security presents an opportunity for Africa to strengthen its diplomatic engagement in this critical area [24]. By collaborating on cross‐border surveillance, pandemic preparedness, and joint responses to health emergencies, African nations can enhance their collective security and attract greater international support for strengthening their health systems [25]. Health diplomacy can be instrumental in developing regional frameworks for health security and fostering partnerships with international actors in this domain.

## 5 Obstacles

Despite the considerable opportunities, health diplomacy in Africa faces a complex array of challenges that can hinder its effectiveness. One significant challenge lies in the diversity of political landscapes and priorities across the continent [26]. Differing national interests, varying levels of political stability, and diverse approaches to governance can complicate efforts to achieve consensus and coordinated action on health issues [27]. Navigating these complex political dynamics requires skillful and sustained diplomatic engagement.

Issues of national sovereignty can also present a considerable challenge to health diplomacy [27]. Although collaboration on cross‐border health threats is essential, some nations may be hesitant to cede perceived authority or control over their national health policies and responses. Building trust and demonstrating the mutual benefits of collective action are crucial for overcoming such sensitivities and fostering a spirit of shared responsibility [27].

Resource disparities among African nations pose another significant challenge. Vast differences in economic capacity and the availability of human and financial resources can create imbalances in the ability of countries to participate in and benefit from regional health initiatives [28]. Health diplomacy must address these disparities through mechanisms for resource sharing, capacity building, and equitable distribution of benefits to ensure that all nations can effectively contribute to and gain from collaborative efforts [3].

Weak institutional capacity at both national and regional levels can also impede effective health diplomacy. Insufficiently resourced and understaffed ministries of health and regional bodies may lack the technical expertise and logistical capacity to effectively engage in complex diplomatic negotiations and implement collaborative health programs [29]. Investing in capacity building for health diplomats and strengthening the institutional frameworks for regional health collaboration is essential.

The influence of external actors and competing interests can also present challenges to Africa's health diplomacy agenda [30]. Although international partnerships are often vital, ensuring that external support aligns with African priorities and does not undermine local ownership and sustainability requires careful diplomatic management [27]. Navigating the complex web of international donors, global health initiatives, and their respective agendas demands strategic engagement to safeguard African interests.

Communication and coordination challenges among the various actors involved in health diplomacy can also hinder progress. Ensuring effective information sharing, aligning strategies, and avoiding duplication of efforts among governments, regional bodies, NGOs, and international partners requires robust communication channels and coordination mechanisms [3]. Strengthening these linkages through diplomatic efforts is crucial for maximizing the impact of collaborative initiatives.

Finally, sustainability and long‐term commitment represent an ongoing challenge. Maintaining political will, securing sustained financial support, and ensuring the long‐term viability of regional health initiatives require continuous diplomatic engagement and a shared vision for the future of health in Africa [27]. Overcoming short‐term priorities and fostering a long‐term commitment to collaborative health action is essential for achieving lasting impact.

## 6 The Way Ahead

The future direction of health diplomacy in Africa must focus on building upon existing prospects while proactively addressing the persistent obstacles. A key priority should be strengthening the capacity of African health professionals and diplomats in the art and science of health diplomacy. Investing in training programs, fostering networks of health diplomats, and embedding diplomatic skills within national and regional health institutions will be crucial for effective engagement and negotiation.

Promoting greater harmonization of health policies and regulations across the continent should be another central focus. Leveraging the platforms of the AU and RECs to develop common frameworks for drug regulation, disease surveillance, and health standards will facilitate cross‐border collaboration and enhance the quality and safety of health interventions.

The future should also see a greater emphasis on leveraging digital technologies to enhance health diplomacy. Developing interoperable digital health platforms for information sharing, telemedicine, and joint research initiatives can overcome geographical barriers and facilitate more efficient collaboration. Diplomatic efforts should focus on establishing data governance frameworks and promoting digital health literacy across the continent.

Fostering innovative partnerships with non‐state actors, including African civil society, the private sector, and philanthropic organizations, will be crucial for unlocking additional resources, expertise, and community engagement in health diplomacy efforts. Creating inclusive platforms for dialogue and collaboration with these actors will be essential.

Strengthening Africa's voice and influence in global health governance is another critical future direction. African nations must continue to coordinate their positions and advocate for their specific needs and priorities in international forums. Building strategic alliances with other developing regions can amplify Africa's impact on global health policies and initiatives.

Finally, ensuring sustainable financing and long‐term political commitment to health diplomacy initiatives is paramount. African leaders must prioritize health as a key pillar of development and security, allocating adequate resources and championing regional health collaborations. Strengthening domestic resource mobilization for health and advocating for sustained international support will be essential for the long‐term success of health diplomacy in Africa.

## 7 Conclusion

Health diplomacy presents a powerful tool for advancing health outcomes and strengthening health systems across the diverse landscape of Africa. The continent possesses significant opportunities to leverage regional collaboration, collective bargaining power, and technological advancements to address shared health challenges. However, realizing the full potential of health diplomacy requires a concerted effort to overcome persistent challenges related to political diversity, sovereignty concerns, resource disparities, and institutional capacity. The future direction of health diplomacy in Africa must prioritize capacity building, policy harmonization, the strategic use of digital technologies, innovative partnerships, and a unified continental voice on the global stage. By embracing a collaborative and strategically oriented approach to health diplomacy, African nations can pave the way for a healthier, more secure, and prosperous future for all their people.

## Author Contributions

Majani Edward conceived the study design, authoring original draft and revising the manuscript.

## Funding

The author has nothing to report.

## Ethics Statement

The author has nothing to report.

## Consent

The author has nothing to report.

## Conflicts of Interest

The author declares no conflicts of interest.

## Data Availability

The author has nothing to report.
